# Effects of Glycine on Collagen, PDGF, and EGF Expression in Model of Oral Mucositis

**DOI:** 10.3390/nu10101485

**Published:** 2018-10-12

**Authors:** Odara Maria de Sousa Sá, Nilza Nelly Fontana Lopes, Maria Teresa Seixas Alves, Eliana Maria Monteiro Caran

**Affiliations:** 1 Department of Pediatrics, Federal University of São Paulo, São Paulo 04023-062, Brazil; 2 Former Head Division of Dentistry, Pediatric Oncology Institute, São Paulo 04023-062, Brazil; nnflopes@terra.com.br; 3 Department of Pathology, Federal University of São Paulo, São Paulo 04023-062, Brazil; mtseixas@patologia.epm.br; 4 Department of Pediatrics, IOP/GRAACC Medical School of Federal University of São Paulo, São Paulo 04023-062, Brazil; elianacaran@terra.com.br

**Keywords:** oral mucositis, glycine, intercellular signaling peptides, proteins

## Abstract

Oral mucositis is frequently a toxic effect of chemotherapeutic and/or radiotherapeutic treatment, resulting from complex multifaceted biological events involving DNA damage. The clinical manifestations have a negative impact on the life quality of cancer patients. Preventive measures and curative treatment of mucositis are still not well established. The glycine has anti-inflammatory, immunomodulatory, and cytoprotective actions, being a potential therapeutic in mucositis. The objective was to evaluate the effects of glycine on the expression of collagen and growth factors, platelet and epidermal in a hamster model oral mucositis. The mucositis was induced by the protocol of Sonis. There were 40 hamsters used, divided into two groups: Group I-control; Group II-supplemented with 5% intraperitoneal glycine, 2.0 mg/g diluted in hepes. Histopathological sections were used to perform the immune-histochemical method, the evaluation of collagen expression, and the growth factors: Epidermal growth factor (EGF) and platelet (PDGF). It was observed that the group supplemented with glycine experienced higher amounts of collagen expression and predominance type of collagen I. The glycine group presented lower immunoexpression of the growth factors, EGF and PDGF. The group supplemented with glycine showed a marked healing process of the oral mucosite, demonstrated by the predominance of collagen type I and reduction of growth factors, EGF and PDGF.

## 1. Introduction

Mucositis is frequently a toxic effect of chemotherapeutic and/or radiotherapeutic treatment, affecting the gastrointestinal tract, mainly the oral cavity. Mucositis is the result of complex and dynamic biological events, involving DNA damage, as well as multiple signaling and interactions between epithelial, connective, and submucosal tissue. Patients with oral lesions may present dysphagia, odynophagia, weight loss, risk of opportunistic agent infections, depression, and low adherence to treatment [1,2]. The pain is usually frequent and intense. Studies evaluating the life quality of patients with head and neck carcinomas, who received radiotherapy, demonstrated the negative impact of mucositis that compromised the physical, functional, emotional, and social domain [3].

On the other hand, reducing the dose/intensity of chemotherapy and/or radiotherapy caused by mucositis, may interfere with treatment effectiveness and cure rates [4]. However, these strategies are not universally accepted, and studies that evaluate the effectiveness of therapeutic regimens present controversial results [5,6].

The use of the amino acid glycine is a new option in the treatment of mucositis. It demonstrates anti-inflammatory, immunomodulatory, and cytoprotective effects in numerous experimental models, such as ischemia, reperfusion, shock, transplantation, alcohol and non-alcoholic hepatitis, fibrosis, arthritis, cancer, toxic drugs, and diabetes [7]. A study in an experimental model showed that glycine supplementation reduced the severity of oral mucositis in multiple aspects: Clinical manifestations, inflammatory process, ulcerations, and lipid peroxidation [8].

It should be noted that glycine supplementation is simple to perform, has a low cost, and does not require an invasive method of treatment. Considering the relevance of the topic and the need for new therapeutic options for the treatment of oral mucositis; the aim of this study was to evaluate the effect of glycine supplementation on collagen and epidermal growth factor (EGF) and platelet (PDGF) expression in hamsters model oral mucositis.

## 2. Materials and Methods

The study was approved by the Ethics Committee of São Paulo Federal University (UNIFESP) (CEUA nº 466174). Forty female Golden Syrian hamsters (Mesocricetus auratus), 8 weeks old, and weighing approximately 150 g each, were used. The animals were kept in groups of six per plastic container, with food and water available ad libitum.

### 2.1. OM Induction Protocol

A well-accepted published protocol for chemotherapy-induced oral mucositis in hamsters was used, as described in Reference [9]. Briefly, all the animals received 80 mg/kg, intraperitoneally, of the chemotherapy drug 5-fluorouracil (EUROFARMA LABORATÓRIOS S.A., São Paulo, Brazil) (5-FU) on day 0, followed by 40 mg/kg 5-FU administered intraperitoneally on day 2. The right cheek pouch of the animals was everted, and the mucosa was irritated by superficial scratching with the tip of an 18-gauge needle by the same operator on days 3 and 4.All the animals were sacrificed with an overdose of anesthetic prior to tissue collection on day 7.

### 2.2. Glycine Supplementation

The animals were randomly divided into two groups of 20 animals each. Animals in Group 1 received a 2.0 mg/g of body weight intraperitoneal injection of Glycine (Ajinomoto, Raleigh, NC, USA), diluted in hepes, at a concentration of 5%. Treatment with the Glycine was initiated on day 0, with application once per day (in the morning), for seven days. Animals in Group 2 served as controls and did not receive any glycine supplementation but were treated identically in all other respects.

### 2.3. Picrosirius Staining for Collagen

Hamster cheek pouch tissue sections (2 mm thick) were subjected to the picrosirius staining protocol, as previously described in Reference [10]. Briefly, sections were deparaffinized and rehydrated, immersed in saturated picric acid (Finoric LLC, Houston, TX, USA) (2 g/100 mL) for 10 min. Sections were then stained for 30 min in a 0.1 per cent solution of Sirius red (0.1 g of Sirius red F3BA (Sigma-Aldrich, St. Louis, MO, USA), in 100 mL of saturated picric acid). This was followed by rinsing with tap water, and re-immersion in picric acid for 10 min. Slides were then dehydrated in absolute alcohol (Sigma-Aldrich^®^, Saint Louis, MO, USA), clarified in xylene (Sigma-Aldrich^®^, Saint Louis, MO, USA), and mounted in synthetic resin (Showa Denko k.k.^®^, Tokyo, Japan). Using polarized microscopy (Olympus, New York, NY, USA), this technique resulted in a red or yellow birefringent appearance of type I collagen.

### 2.4. Quantitative Collagen Analysis

The quantitative analysis of collagen was performed using an BX51P polarizing microscope (Olympus, New York, NY, USA) with a plan achromatic objective, coupled to a camera (Sony CCD-Iris, model DXC-107ª, Tokyo, Japan) and a PC, including a Pentium 233MMX processor and a plate scanner. Images were analyzed in the software CorelDraw Graphics Suite X4 (version 12, Ottawa, ON, Canada) and measured interactively with the original image using the software Image Tool (version 3.00, developed University of Texas Health Science Center, San Antonio, TX, USA (UTHSCSA)) for Windows, available freely online from the University of Texas Health Science Center at San Antonio. The image was evaluated in greatest staining (“hot spot”) in the tissue sections, at ×400 magnification. Collagen staining was quantified as the percentage ratio of the area of staining in the studied image, as compared to the total area of the digitized field. For qualitative assessment, collagen was scored by a blinded examiner as mature collagen (well organized and thick collagen fibers) or immature collagen (loosely dispersed and poorly organized thin collagen fibers).

### 2.5. Immunohistochemical Technique

Heat-induced epitope retrieval was used for factor EGF and PDGF (30 min in a microwave). For all antigens, endogenous peroxidase was quenched by immersion in 3% hydrogen peroxide, five times, for 5 min each. Monoclonal mouse primary antibodies (Abcam^®^, Burlingame, CA, USA) for EGF (1:200), and PDGF (1:100), were applied and incubated overnight at 4 °C. The slides were then rinsed in phosphate buffered saline (PBS) (Sigma-Aldrich^®^, Saint Louis, MO, USA) for 30 min, and after that, incubated with biotinylated goat anti-rabbit secondary antibody (Jackson Immuno Research, West Grove, PA, USA) for 15 min at 37 °C, rinsed again in PBS, and incubated for 15 min in avidin-biotin-peroxidase complex (StreptABCcomplex-HRP, Dako, Glostrup, Denmark). The final reaction was achieved by incubating the sections with 3,3′-Diaminobenzidine tetrahydrochloride hydrate (Sigma-Aldrich^®^, Saint Louis, MO, USA) in the presence of 0.05% hydrogen peroxide (Sigma-Aldrich^®^, Saint Louis, MO, USA) in PBS, for 5 min at 37 °C. Slides were then rinsed with tap water, counterstained with Harris’ hematoxylin protocol (Sigma-Aldrich^®^, Saint Louis, MO, USA) for 5 min, rinsed in ammonia water (Sigma-Aldrich^®^, Saint Louis, MO, USA), dehydrated, and coverslipped. As positive tissue controls, identical procedures were performed for mouse mamacarcinoma (EGF and PDGF).

### 2.6. QuantitativeAnalysis of EGF and PDGF

The EGF and PDGF were quantified in epithelial staining, in two microscopic fields, at ×400 magnification, beginning in greatest staining (“hot spot”) in the given sample. The images were digitized and analyzed in the software CorelDraw Graphics Suite X4 (version 12, Ottawa, ON, Canada) and measured interactively with the original image, using the software Image Tool (version 3.00, developed University of Texas Health Science Center, San Antonio (UTHSCSA)). Staining was quantified as a percent ratio, between the area of immune labeling in the studied image and the total area of the digitized field.

## 3. Results

### 3.1. Quantitative and Qualitative Analysisof Collagen Expression

**Figure 1:**
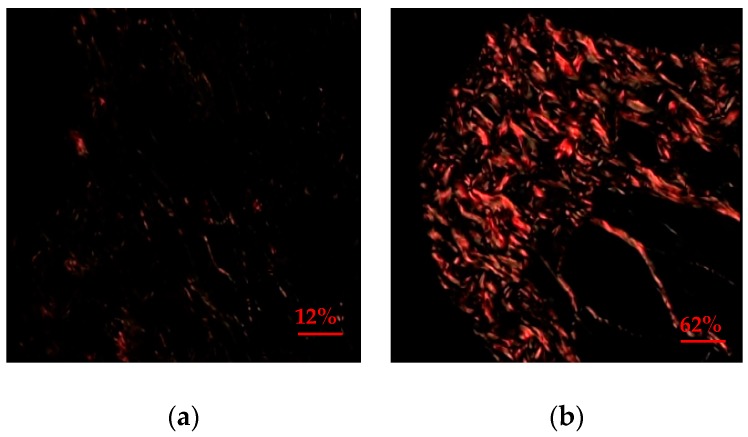
Photomicrography of the expression of collagen in jugal mucosal of hamster obtained by picrossirius method under polarized light, in oral mucositis model at day 7, increase of 400×. (**a**) Photomicrography of the jugal mucosal, type III collagen, type 2, delicate and greenish fibers in the animals of the control group. (**b**) Photomicrography of the jugal mucosal, demonstrating type I collagen, thick and yellow-orange in animals of group supplemented with glycine.

In relation to the quantitative evaluation of the collagen percentage, the mean of the group supplemented with glycine was higher, with a statistically significant difference in relation to the control group (*p* = 0.002) (Table 1). The group supplemented with glycine showed a greater amount of collagen type I, 95% (*n* = 19) on day 7, when compared to the control group. In contrast, only 5% (*n* = 1) of the control group had collagen type I on day 7 (Table 2 and Figure 1).

### 3.2. Quantitative Evaluation of Epidermal Growth Factor (EGF)

**Figure 2:**
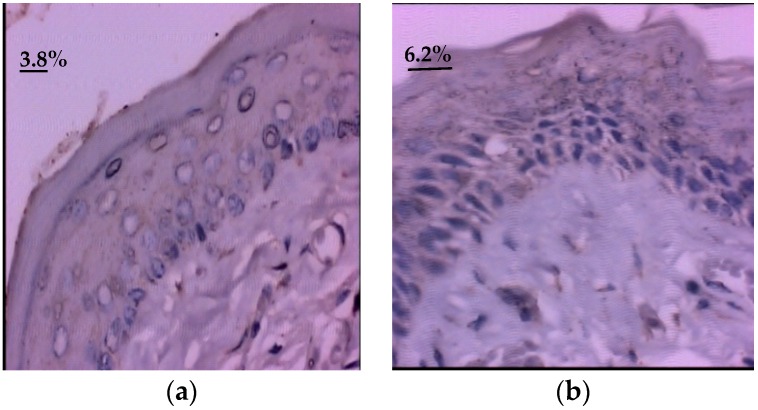

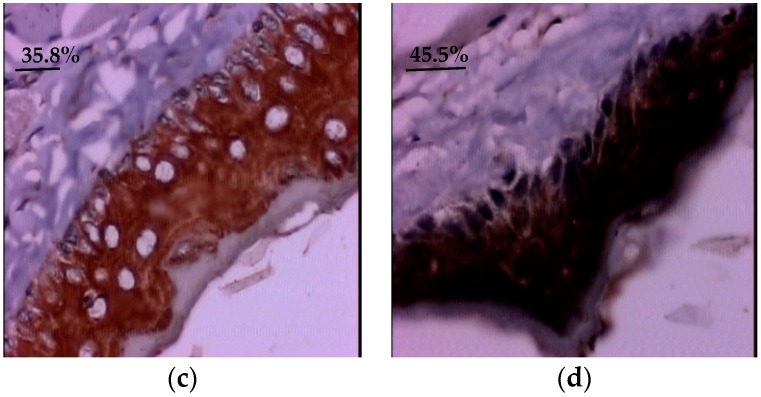
Photomicrograph of epidermal growth factor (EGF) immunoexpression in the oral mucosal of hamster with oral mucositis induced by 5-Fluorouracil (5-FU), increased by 400×. (**a**,**b**) Group supplemented with glycine showed lower EGF immunoexpression, when compared to the control group (**c**,**d**) an increase in epidermal growth factor expression was observed.

In group II, there was a statistically significant reduction in the EGF mean, compared to the control group (*p* = 0.001) (Table 3 and Figure 2). The EGF immunoexpression in the jugal mucosal of the group hamsters of the glycine-supplemented was lower, when compared to the control group.

### 3.3. Quantitative Evaluation of Platelet Derived Growth Factor (PDGF)

**Figure 3:**
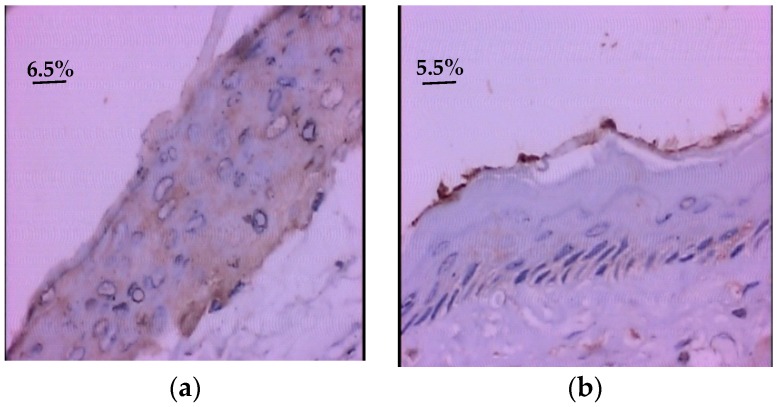

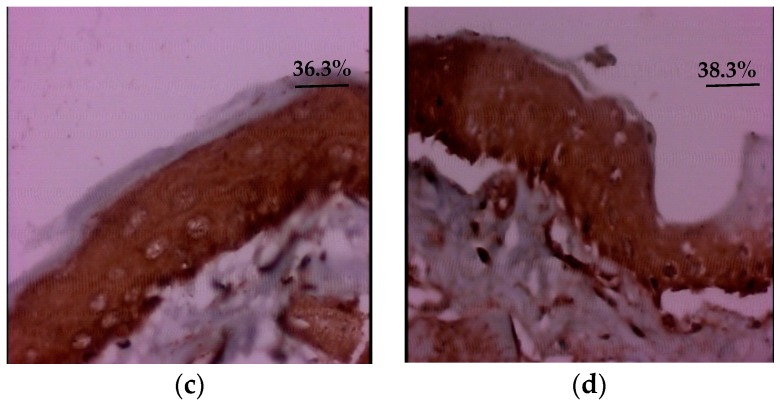
Photomicrograph of the platelet (PDGF) immunoexpression in the oral mucosa of hamster with oral mucositis induced by 5-FU nodia 7 (400×). (**a**,**b**) Group supplemented with glycine showed less immunoexpression of the platelet derived growth factor. (**c**,**d**) In the control group, greater PDGF immunoexpression was observed, when compared to the group supplemented with glycine.

In the glycine-supplemented group, a statistically significant reduction in PDGF immunoexpression was observed when compared to the control group (*p* = 0.001) (Table 4 and Figure 3).

### 3.4. Correlation between Collagen Fiber Types and Amount of Collagen and Immunoexpression Growth Factors: EGF and PDGF

**Figure 4:**
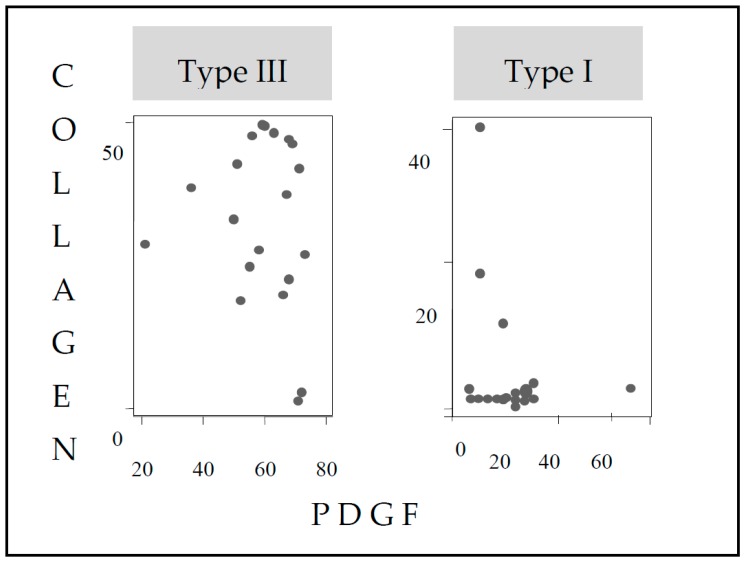
Correlation between collagen fibers (type I and type III) and quantitative collagen and immunoexpression of PDGF, independent of the group.

No statistically significant correlations were observed between the amount of collagen and the immunoexpression of growth factors: EGF and PDGF, among groups studied. However, there was a statistically significant positive correlation (*r* = 0.0293 and *p* = 0.0003), between the amount of collagen and the immunoexpression of PDGF in group I. The correlations between collagen fiber types, amount of collagen, and immunoexpression of growth factors, EGF and PDGF, were not statistically significant. However, there was a correlation between type I collagen, independent of the group, and a statistically significant negative correlation between the amount of collagen and the expression of PGDF (*r* = −0.4421, *p* = 0.05). On the other hand, the expression of type III collagen, independent of the group, showed a positive correlation between the amount of collagen and the immunoexpression of PDGF (*r* = 0.6180, *p* = 0.0036) (Figure 4).

## 4. Discussion

Currently, the acute side effects of antineoplastic treatment, such as anemia, neutropenia, nausea and vomiting, are relatively well controlled with effective and validated support therapies. However, mucositis, although frequent and interfering with the quality of life and therapeutic planning of the cancer patient, lacks effective prophylactic and therapeutic measures [11]. In this context, knowledge about the inflammatory, repair and regeneration processes involved in the pathophysiology of mucositis, is fundamental for proposals of therapies that act on specific receptors or molecules of the complex mucositis mechanism.

Much of the knowledge about the sequence of events occurring in the immune response and regeneration of tissue lesions comes from experiments with hamsters, which are low-cost and widely used models. The hamster jugal that functions as a food reserve has similarities to the human oral mucosa, both in the development of chemotherapy-induced mucositis, and in bacterial flora and blood cells [12,13]. In a previous publication Sá et al., 2013 [8], the authors evaluated clinical and laboratory aspects of hamster jugal mucositis in the initial phase and on day seven. On the third day, all the animals had severe mucositis: Erythema, edema, and ulcers. On the seventh day, the group supplemented with glycine presented clinical and laboratory resolution (i.e., absence of inflammatory infiltrate on histological examination and reduction of lipid peroxidation) (*p* < 0.001), indicating the cytoprotective and anti-inflammatory effect of glycine in the oral mucosa.We studied the effect of glycine supplementation on the amount, types of collagen, epidermal growth factors (EGF), and platelet derivative (PDGF) in experimental mucositis.

Regarding the quantification of collagen, the group supplemented with glycine had a higher quantitative expression of collagen, in relation to the control group. It is important to note that collagen is composed of amino acids, mainly glycine and proline, and is produced by numerous cell lines, such as fibroblasts, chondrocytes, osteoblasts, epithelial, and muscular cells [14,15]. The greater amount of collagen expresses an acceleration in tissue repair, since collagen is the main structural component in wound healing, being fundamental to the resistance and integrity of all tissues [16]. In experimental studies with dietary supplementation of collagen precursor amino acids, such as 0.5–2% glycine [17] and 1% proline [18], there was an increase in height of intestinal villi and absorption of nutrients, as well as weight gain and collagen production. Likewise, the 0.2% glycine supplementation to a protein diet for broilers of 5–21 days increased skeletal muscle growth, type I collagen production, and the efficiency of nitrogen retention [19]. In another animal model study, dietary supplementation with 0.5% glycine improved weight gain, anti-oxidative capacity, and immunity [20]. In vitro studies have shown that adequate supply of glycine and proline are essential for maximum collagen synthesis, and maximum growth performance [21,22,23].

Type I collagen fiber was predominant in the group supplemented with glycine (95%), in relation to the control (5%), where type III fiber predominated. These findings can be explained through studies in the literature, which infer that in the early stages of the healing process, the first collagen fibers deposited at random are type III, which during remodeling are reorganized and replaced by type I collagen, providing greater tensile strength, so that in the more advanced stages of healing there is thicker and more resistant collagen [24,25].

On the other hand, tissue remodeling depends on proteinases released from inflammatory and mesenchymal cells, such as metalloproteinases (MMP), which act little on normal skin, but are extensively recruited to the need for proteolysis. The homeostasis between synthesis and collagen lysis, depends on the simultaneous action of MMP and its non-specific and specific inhibitors. The imbalance between MMP and inhibitors, leads to delayed repair and abnormal resolution of healing. In oral lesions, stromal cells, such as fibroblasts or inflammatory cells, produce matrix metalloproteinases (MMPs) and cytokines (interleukin-1β (IL-1β) and tumor necrosis factor alpha (TNF-α)), which amplify the inflammatory response and induce the activity of collagenases, contributing to acceleration in synthesis and loose distribution in such areas [26]. In our study, the increased amount of collagen and the predominance of type I fiber in the glycine group, suggested that glycine induced acceleration of the tissue repair phase. This finding is relevant because it indicated that animals supplemented with glycine are in advanced stages of healing, and glycine can be directly linked to the organization and remodeling of collagen fibers.

Our results indicated that in the group supplemented with glycine, the expression of epidermal growth factor (EGF) and platelet derivative (PDGF) were significantly reduced, in comparison with the control group, which presented high expression of these factors on the seventh day of the experiment. Growth factors are signal peptides that play a central role in the processes of tissue regeneration and healing. They interact with cell surface receptors, enhancing gene transcription and protein synthesis. The synthesized proteins trigger cellular proliferation and differentiation, besides stimulating the production of extracellular matrix and angiogenesis, favoring the process of tissue repair. The growth factor TGF-β and PDGF stimulate the synthesis of collagen by fibroblasts, while glucocorticoids inhibit its synthesis [27,28]. PDGF is released in the coagulation process and in the mechanism of platelet adhesion, in injury to blood vessels.

In addition, platelets can be produced by monocytes, macrophages, fibroblasts, endothelial cells, bone matrices, and smooth muscle cells, with the function of recruiting neutrophils and monocytes early in healing and promoting the synthesis of collagen and proteoglycans. PDGF leads to increased inflammatory response through chemotaxis to neutrophil inflammatory cells and macrophages and fibroblasts, increasing the deposition of extracellular matrix, besides stimulating the synthesis of DNA and proteins, and acting as a systemic or local regulator, with the potential to achieve rapid cellular settlement and consequently, faster repair.

Fibroblast growth factor (FGF) has chemotactic and mitogenic action for fibroblasts, which initiate collagen production, as well as TGF-β, which also stimulates collagenization. Epidermal growth factor (EGF) leads to acceleration of epithelization, whereas FGF and PDGF stimulate wound contraction and remodeling, during the last stage of the healing process [29,30]. Authors measured the presence and level of EGF in the oral secretion of patients who received radiotherapy in the head and neck and suggest that there is a reduction of the growth factor during treatment. The reduction of EGF was directly associated with increased frequency and severity of mucositis [31]. In the study of Sonis et al., 1992, with hamster-induced mucositis, EGF administration was related to increased severity and duration of mucositis. On the other hand, some evidence related EGF as a key inducer of angiogenesis, invasiveness of tumor cells in—in vitro and in vivo studies—with cells obtained from mouse mammary cancer. Studies have shown that the mRNA levels of this growth factor and its receptors, are reciprocally expressed in mammary carcinoma cells that arise in transgenic mice with a tendency to cancer, especially breast and prostate cancer [32,33]. Glycine in research using animal models of malignant neoplasms, has demonstrated EGF receptor inhibitory action with antiangiogenic and cytoprotective effect [34,35].

Correlation was observed in the control group, between the low amount of collagen and the high immunoexpression of PDGF. In addition, there were correlations between the types of fibers (type I and III) and the amounts of collagen and PDGF immunoexpression. Type I fibers have the highest total amount of collagen and the lowest PDGF immunoexpression. In the collagen III fibers, there is a similar amount of collagen and a greater immunoexpression of PGDF. An immunoexpression of PDGF growth factor correlates with the healing process and fiber remodeling. The increased immunoexpression of PDGF is associated with the lower amount of collagen and fine fibers. On the other hand, the reduction of the PDGF immunoexpression correlates with the increase of the total amount of collagen, as well as the presence of thick type III fibers. The PDGF growth factor seems relevant in the healing process, through the remodeling of the types of collagen fibers. As there is the remodeling of type III to type I fiber, there is a reduction in the quantitative expression of PDGF.

In our study, the data suggested that the group supplemented with glycine at day 7 had lower cell signaling, hypothetically this may be associated with lower inflammatory stimulus, or simultaneous with cytoprotective effects generated by glycine against cellular apoptosis. The design of the experiment limits the conclusive analysis, since the data were evaluated only on day 7, end of the experiment, it would be important to describe the effect of glycine on the expression of growth factors in the initial period.

Comparing the results of glycine supplementation with studies using low intensity laser therapy in the treatment of oral mucositis induced by chemotherapy, similar results were observed, i.e., accelerated tissue repair, elevated expression of type I collagen, and Cyclooxygenase (COX)-2 reduction, and of the vascular endothelial growth factor (VEGF), promoting the faster healing of oral mucositis lesions [36].

Glycine supplementation has been shown to have effects on cell signaling and proliferation, and its use in mucositis is reinforced by the presence of glycine receptors in gingival tissues and oral keratinocytes [37]. Our intervention was carried out in the experimental framework, a model that is easy to reproduce and is reliable [9].

The evaluation of the effect of glycine at different times of the development of mucositis could elucidate its action on growth factors and mechanism of action. It is also relevant to continue evaluations on the effect of glycine on other oral mucositis markers, such as metalloproteinases and expressions of genes. Studies point to the safety of supplementation of this amino acid in humans [38]. However, extrapolations to the clinic should be prudent, through studies duly controlled by protocols authorized by ethics committees, contributing to the adoption of preventive and therapeutic protocols.

This study is the first to point out that supplementation of glycine in the oral mucositis model raises the expression of type I collagen, and quantitatively reduces the growth factors, EGF and PDGF. These data suggest that glycine supplementation alters the process of signaling, cell proliferation, cicatrization and cellular reepithelialization, relevant phases in oral mucositis.

## 5. Conclusions

The results obtained under the conditions of this experimental study suggested that the glycine supplementation at 0.2 mg/g in hamster-induced oral mucositis during a 7-day period, favors the cellular healing process, increasing collagen synthesis, and accelerating the remodeling of collagen fibers, predominance of thick fibers, collagen type I. The group supplemented with glycine showed lower immunoexpression of epidermal growth factors (EGF) and platelet derivative (PDGF), in the final period of the experiment. The reduced concentration of cellular signals is probably associated with the advanced stage of the healing process. The type of collagen fiber (type I and III), regardless of the group studied, showed an inverse correlation between the amount of collagen and the derived growth factor (PDGF). This correlation was probably associated with the PDGF fiber remodeling function. Experimental models of oral mucositis are of great importance for the clarification of the mechanisms of action of glycine, contributing to the development of new therapeutic approaches related to anticancer therapy.

## Figures and Tables

**Table 1:** Quantitative evaluation of total collagen expression in hamster-induced oral mucositis.

Group	Quantitative Evaluation of Collagen Expression (%)						
	Mean	Medium	SD	IC 95%	Minimum	Maximum	Valor *p*
Group I (Control)	25.0	25.3	3.8	23.2–26.8	20.4	30.4	0.0002 *
Group II (Glycine)	61.6	63.2	9.4	57.2–66.0	36.2	73.2	

SD: Standard deviation; IC 95%: Confidence Interval; * Wilcoxon-Mann-Whitney test.

**Table 2:** Qualitative evaluation of collagen expression in hamster-induced oral mucositis.

Group	Qualitative Evaluation of Collagen Expression		
	Type I N (%)	Type III N (%)	Valor *p*
Group I (Control)	1 (5)	19 (95)	0.0001 *
Group II (Glycine)	19 (95)	1 (5)	

* Qui-quadrado test.

**Table 3:** Quantitative evaluation of epidermal growth factor (EGF) immunoexpression in hamster-induced oral mucositis.

Group	Quantitative Evaluation of EGF Immunoexpression (%)						
	Mean	Medium	SD	IC 95%	Minimum	Maximum	Valor *p*
Group I (Control)	40.3	40.2	11.4	34.9–45.7	9.4	65.4	0.0001 *
Group II (Glycine)	7.3	6.5	7.2	3.9–10.7	1.9	35.5	

SD: Standard deviation; IC 95%: Confidence Interval; * Wilcoxon-Mann-Whitney test.

**Table 4:** Quantitative evaluation of platelet derived growth factor (PDGF) immunoexpression in hamster-induced oral mucositis.

Group	Quantitative Evaluation of PDGF Immunoexpression (%)						
	Mean	Medium	SD	IC 95%	Minimum	Maximum	Valor *p*
Group I (Control)	31.3	35.1	16.2	23.7–38.9	1.3	49.6	0.0001 *
Group II (Glycine)	10.8	1.5	6.4	1.3–11.5	1.1	40.8	

SD: Standard deviation; IC 95%: Confidence Interval; * Wilcoxon-Mann-Whitney test.
